# The disease course in microscopic colitis may be influenced by hormonal factors

**DOI:** 10.1186/s12876-025-04083-8

**Published:** 2025-06-19

**Authors:** Klas Sjöberg, Lina Vigren, Marie-Rose Mellander, Izabella Janczewska, Hans Strid, Elisabeth Hultgren Hörnquist, Andreas Münch

**Affiliations:** 1 Department of Clinical Sciences, Lund University, Malmö, Malmö, Sweden; 2 Department of Gastroenterology and Nutrition, Skåne University Hospital, Malmö, Sweden; 3 Capio Gastro Center Skåne, Lund, Sweden; 4 Centre for Digestive Health, IBD Unit, Stockholm University Hospital, Stockholm, Sweden; 5 Department of Gastroenterology, Ersta Hospital, Stockholm, Sweden; 6 Gastroenterology unit, Centre for Digestive health, Department of Gastroenterology, Dermatovenereology and Rheumatology, Karolinska University Hospital, Stockholm, Sweden; 7 School of Medical Sciences, Örebro University, Örebro, Sweden; 8 Department of Gastroenterology and Hepatology, Linköping University, Linköping, Sweden

**Keywords:** Contraceptives, Estrogen, MHT, Microscopic colitis, Oophorectomy, Sex hormones

## Abstract

**Background:**

Microscopic colitis (MC) is characterized by non-bloody, watery diarrhea predominantly in elderly women. Known risk factors are smoking, medication with NSAIDs, PPIs or SSRIs, while data on hormonal factors is sparse. The aim of the present study was to investigate whether hormonal factors that disrupt the sex hormonal balance could have an impact on the disease course in MC.

**Methods:**

A questionnaire was distributed to 384 women with microscopic colitis (MC) (mean age 64 years, range 35–90) from five centers in Sweden about demographic aspects including age at diagnosis, disease duration, treatment, and polycystic ovary syndrome, endometriosis, bilateral oophorectomy, previous or ongoing medication with hormones or in vitro fertilization (IVF) in relation to the disease course.

**Results:**

The association with smoking could be verified. In relation to the disease course the odds ratio (OR) was higher for celiac disease and oral contraceptives but lower for hormone replacement therapy but for the two latter non-significant. However, bilateral oophorectomy had a significantly lower OR (0.41, CI 0.19–0.86, *p* = 0.019). No other factors had any substantial impact on the disease course.

**Conclusion:**

An association was verified with smoking. Celiac disease may be associated with more active disease. The observed lower OR for more active disease after bilateral oophorectomy is in line with a previously suggested association between the risk of MC and the hormonal balance. The exact mechanisms behind the hormonal effect on the disease course found in the present study are although still obscure.

**Supplementary Information:**

The online version contains supplementary material available at 10.1186/s12876-025-04083-8.

## Introduction

Microscopic colitis (MC) is a chronic, inflammatory bowel disease with watery, non-bloody diarrhea predominantly affecting elderly women. Since the diagnosis is based on typical histological alterations the term MC is logical [1]. It is divided into collagenous colitis (CC) presenting with a thickened subepithelial collagen band and lymphocytic colitis (LC) which is characterized by an increased number of intraepithelial lymphocytes. The pathogenesis is obscure, but MC is generally considered to be an immune mediated disease. Especially in the subtype collagenous colitis an association with HLA DQ2 has been found [2]. A barrier dysfunction, as well as an increased number of intraepithelial T lymphocytes in combination with a prompt response after treatment with local steroids also indicate that the disease is immune driven [3]. However, the criteria for classical autoimmunity are not fulfilled, e.g., auto-antibodies have not yet been identified [4].

Among risk factors smoking, female gender and age are established. High alcohol intake [5], GI infections [6] and medication (NSAIDs, PPIs or SSRIs) are associated with enhanced risk for disease [7, 8].

Hormonal changes affect immune function and the disease onset and course of immune driven diseases. Pregnancy influences mucosal barrier function and induces immune tolerance based on hormonal alterations, illustrating the impact of hormonal modifications in inflammatory bowel disease (IBD) [9]. Female sex hormones, especially estrogen, are associated with many autoimmune and immune driven diseases, and especially SLE has been studied extensively in this respect [10]. When it comes to IBD, previous studies have shown an association between the use of oral contraceptives and enhanced risk of Crohn`s disease (CD), and the use of menopausal hormone therapy (MHT) and increased risk of UC. These associations are thought to be mediated by alterations in permeability, a shift in the T helper cell subsets balance and changes in the gut microbiota [11].

In view of the observations in IBD, with an increased risk after hormonal alterations, the possibility that hormones could impact the risk for MC has been contemplated. Furthermore, the clear predominance of postmenopausal women has been confirmed in numerous epidemiological studies raising the question if hormones are involved in MC pathogenesis. One study administered a questionnaire to 131 women with MC and compared this group with a cohort consisting of 737 controls. The questionnaire included questions about parity, age at first childbirth, menarche and menopause, the use of oral contraceptives, and MHT. More patients than controls had been exposed to oral contraceptives at any time, but fewer were on current MHT. However, neither use of oral contraceptives, current MHT or previous bilateral oophorectomy had any impact on disease activity [12]. Patients with lymphocytic colitis had given birth less often compared to those with collagenous colitis (OR = 0.20), however no differences were observed between patients with persistent or transient disease. MC patients were more seldom older than 15 years of age at menarche (OR = 0.48) and were younger at menopause (OR = 0.30) compared with controls. Even though some factors could indicate a hormonal influence, no obvious association between factors influencing sex hormone levels and presence of MC could be found in that study [12].

In contrast, a study based on data from 227,766 women who participated in the Nurses’ Health Study (NHS) and the NHSII identified 275 incident cases of microscopic colitis. The findings revealed that current use of MHT was associated with an increased risk of MC, with a multivariable-adjusted hazard ratio of 2.64. The risk increased with longer duration of use (P for trend < 0.0001) and decreased after discontinuation (P for trend = 0.002). Furthermore, ever use of oral contraceptives was associated with increased risk of MC (multivariable-adjusted hazard ratio 1.57). However, there were no associations between age at menarche, parity, age at first childbirth, age at menopause, or induced menopause type and incident MC [13]. While the first mentioned study [12] did not reveal any differences between patients with persistent or transient disease regarding the use of oral contraceptives, HRT or oophorectomy, the second study did find an increased risk of MC after exposure for HRT [13]. However, this latter association was observed for disease onset and not disease course as in the former. Thus, the results are contradictory and as an association with hormones seems apparent in other immune driven diseases, investigations in MC with other designs are warranted.

The aim of the present study was to investigate whether hormonal factors, such as diseases, surgery (bilateral oophorectomy) or medications that disrupt the hormonal balance could have an impact on disease course in MC.

## Patients and methods

### Patients

Patients with MC were randomly recruited from five hospitals in Sweden: Karolinska university hospital, Ersta hospital, Stockholm, Södra Älvsborg hospital, Borås, Linköping university hospital, and Skåne university hospital (located in Malmö and Lund). They were identified in the health care registries by the Systematized Nomenclature of Medicine (SNOMED) system for biopsies at the respective Departments of Pathology to identify all patients with LC and CC through their specific codes (M40600 for CC and M47170 for LC). The validity of MC cases in the Swedish Pathology registries has been evaluated with a positive predictive value of 95% [14].

### Questionnaire

A questionnaire was sent to female patients attending the out-patient clinic at the respective hospitals. Data on demography, hormonal factors, and presence of relevant diseases can be seen in Table 1.

### Statistics

The impact of hormonal factors on the disease course was determined with logistic regression. The outcome variable “disease course” was divided into four groups: Quiescent disease (1), remitting disease (2), remitting disease with requirement for continuous treatment (3) and chronic, active disease (4). Two rounds of calculations were carried out, first one where 1 was compared to 2, 3 and 4 and then one where 1 and 2 were compared to 3 and 4. To avoid a negative impact of missing data in the statistical analysis only those 236 of the 384 participants that had given information about all the factors used in the statistical calculations were included. Statistical analysis was performed using SPSS version 27. A p-value < 0.05 was considered significant.

### Ethics

This study was approved by the Swedish Ethical Review Authority 2019–06338. Written informed consent was obtained from all participating patients and controls before inclusion. All methods used in the study were carried out in accordance with relevant guidelines and regulations.

## Results

### Patients and disease course

In total, 384 women with MC answered the questionnaire (mean age 64 years, range 35–90). The mean age at diagnosis was 59 years, range 13–82 (Table 1). The cohort consisted of elderly women where 1/3 were current (15%) or previous (19%) smokers and 6% had celiac disease, which is in line with previous reports [15].

The disease course was stated in 311 patients, of which 99 (32%) had quiescent disease, 106 (34%) had remitting, 58 (19%) had remitting disease with requirement for continuous treatment and 48 (15%) had chronic, active disease.

**Table 1:** Demographic and hormonal characteristics of the patients with microscopic colitis

		MC		CC		LC	
Total (N)		384		210		174	
Age (years)	Mean (range)	69	(35–90)	69	(41–90)	70	(35–87)
Age at diagnosis	Mean (range)	59	(13–82)	58	(18–82)	60	(13–80)
BMI	Mean (range)	25	(15–41)	25	(16–38)	25	(15–41)
Smoking (%)	Current (%)	57	(15)	32	(15)	25	(14)
	No (%)	252	(66)	144	(69)	108	(62)
	Previous (%)	73	(19)	32	(15)	41	(24)
Celiac disease	(%)	25	(6.5)	14	(6.7)	11	(6.3)
Menarche (age)	Mean (range)	13	(9–18)	13	(9–18)	13	(10–17)
Menopaus (age)	Mean (range)	49	(36–45)	49	(36–60)	49	(37–65)
Children (N)	Mean (range)	2.2	(0–5)	2.2	(0–5)	2.1	(0–5)
Diseases	PCOS (%)	6	(1.6)	3	(1.4)	3	(1.7)
	Endometriosis (%)	33	(8.6)	20	(9.5)	13	(7.5)
Oophorectomy	Bilateral (%)	135	(35.2)	65	(31.0)	70	(40.2)
Medication	Oral contaceptives (%)	259	(67.4)	140	(66.7)	119	(68.3)
	HRT (%)	182	(47.4)	91	(43.3)	91	(52.3)
	IVF (%)	18	(4.7)	7	(3.3)	11	(6.3)

MC = microscopic, CC = collagenous and LC = lymphocytic colitis, PCOS = polycystic ovary syndrome, HRT = hormone replacement therapy, IVF = in vitro fertilization

### Factors influencing the hormone balance

The hormonal factors are stated in Table 1. In total 286 persons had used contraceptives whereof 259 (67%) had used oral contraceptives, 22 (8%) intrauterine device, 3 (1%) injection, 1 subcutaneous implant (0.3%) and 65 (23%) other type(s) of contraceptives. Since the hormonal influence from these different treatments differs regarding the hormonal doses and thus impact on disease course only oral contraceptives have been included in the statistical calculations. Endometriosis had been diagnosed in 33 patients (8.6%), As much as 135 patients (35.2%) had gone through bilateral oophorectomy. Menopausal hormone therapy (MHT) had been given to 123 (32%) in the past and 59 (15.4%) had ongoing treatment. Due to small groups and missing values intake of previous and present HRT was merged into one group.

Whether oophorectomy had been carried out uni- or bilaterally was double checked in the patient files in the Malmö and Linköping cohorts consisting of 232 of the patients of which 19 had undergone surgery. Their statement about surgeries proved to be correct in all cases except two. Unfortunately, the patients could not remember for how long they had been treated with MHT or contraceptives, neither could they state the time point when they had gone through surgery.

### The effect of hormonal factors on the disease course

The outcome variable “disease course” was thus as stated above divided into four groups: Quiescent disease (1), remitting disease (2), remitting disease with requirement for continuous treatment (3) and chronic, active disease (4). Two rounds of calculations were carried out, one where 1 was compared to 2, 3 and 4 and one where 1 and 2 were compared to 3 and 4. When comparing quiescent disease with the other three more aggressive disease courses no significant results could be seen (data not shown). The results from the comparison between quiescent and remitting disease versus remitting disease with requirement for continuous treatment and chronic, active disease are stated below.

In the logistic regression the putative associations were determined in blocks. First the impact of oral contraceptives and HRT but also smoking and celiac disease were taken into consideration. In the next step BMI, age at diagnosis and childbirth (i.e., given birth to any child vs. no children) were included as well (adjusted for smoking and celiac disease). Finally, endometriosis, bilateral oophorectomy and IVF were also included (adjusted for smoking and celiac disease) while PCOS had to be excluded due to too few cases (*N* = 6).

Based on the results in Table 2a the intake of oral contraceptives showed a trend for increased odds ratio (OR) for a more severe disease course while the opposite was the case for HRT. However, none of these comparisons reached any significance. If the numbers are too small or if there is no association is not possible to determine. However, smoking and celiac disease had a significant impact, especially ex-smoking.

In Table 2b, just as in Table 2a, smoking and celiac disease increased the OR, ex-smokers more than current smokers while the association with celiac disease did not reach significance in this calculation although a trend could be seen.

In Table 2c the risk of ex-smoking was even more profound. Endometriosis and IVF did not influence the disease course while bilateral oophorectomy seemed to be protective.

Due to the low numbers no calculations have been caried out with subtypes of MC.

**Table 2:** Factors influencing the disease course in microscopic colitis

Table 2a	p	OR	95% C.I. for OR	
Oral contraceptives	0.313	1.41	0.72–2.74	
Current and/or previous treatment with HRT	0.152	0.66	0.37–1.17	
Smoking	0.016			
Ex-smoker	0.005	2.85	1.36–5.95	
Current	0.192	1.67	0.77–3.60	
Celiac disease	0.049	2.70	1.00–7.29	
Table 2b	p	OR	95% C.I. for OR	
Oral contraceptives	0.535	1.24	0.62–2.49	
Current and/or previous treatment with HRT	0.211	0.69	0.38–1.24	
Smoking	0.020			
Ex-smoker	0.007	2.80	1.33–5.91	
Current	0.210	1.64	0.76–3.58	
Celiac disease	0.073	2.50	0.92–6.82	
BMI	0.246	0.96	0.91–1.02	
Age at diagnosis	0.933	0.99	0.97–1.03	
Children	0.234	1.81	0.68–4.81	
Table 2c	p	OR		
			95% C.I. for OR	
Oral contraceptives	0.602	1.21	0.59–2.46	
Current and/or previous treatment with HRT	0.354	0.76	0.42–1.37	
Smoking	0.002			
Ex-smoker	0.001	5.07	2.03–12.68	
Current	0.169	1.74	0.79–3.85	
Celiac disease	0.115	2.28	0.82–6.35	
BMI	0.164	0.96	0.90–1.02	
Age at diagnosis	0.944	0.99	0.97–1.03	
Children	0.251	1.79	0.66–4.83	
Endometrios	0.687	0.80	0.28–2.33	
Oophorectomy (bilateral)	0.019	0.41	0.19–0.86	
IVF	0.856	0.86	0.18–4.20	

The letters a, b and c refer to the different blocks in the logistic regression. HRT = hormone replacement therapy

## Discussion

In the present study intake of oral contraceptives or HRT did not have any significant impact on the disease course. In contrast, smoking and celiac disease increased the risk for more active disease, something that had been observed before at least for smoking [15–17]. The factors BMI, age at diagnosis or childbirth were not associated with the disease course. Among the conditions affecting the hormone balance endometriosis and IVF were not associated while oophorectomy seems to have a protective effect.

The lack of association with oral contraceptives or HRT could indicate that there is no actual effect. The p-values are far from significant. Furthermore, the risk ratios are contradictory. It is not possible to draw any conclusions from the results in the present study. However, a previous study from 2013 found that more patients than controls had been exposed to oral contraceptives but fewer to current MHT, but without any effect on disease activity [12]. This is in line with our observed risk ratios. A latter and larger study from 2018 studied the risk for disease onset in relation to use of hormone replacement therapy and found it to be highest in patients on current therapy [13]. The finding that oral contraceptives, but not menopausal hormone therapy (MHT), appear to have an impact may seem contradictory at first. However, this discrepancy might be explained by the longer duration of contraceptive use, which typically begins earlier in life. The earlier initiation and prolonged exposure to contraceptives may have played a significant role in influencing the observed outcomes.

The association with smoking was not unexpected. The effect on disease course by smoking has already been proven [15, 16]. However, the association between celiac disease and more active disease has not, to the best of our knowledge, been presented before. Microscopic colitis and celiac disease share the same genetical predisposition, something that could contribute to this phenomenon [18]. Patients with CC have HLA DQ2 to a larger extent than patients with LC and they also have a more severe disease course. It could be that patients with HLA DQ2 risk both to develop celiac disease and a at the same time a more active disease, but this remains to be proven.

Among the other factors influencing the hormone balance, i.e., endometriosis, IVF and oophorectomy a negative association was apparent for the latter. This is in accordance with the hypothesis that the hormone balance could have an impact. A reduction of hormonal influence could consequently influence the disease course towards a more quiescent state,

The study from Burke et al. reported an increased risk for MC *per se* by hormone substitution. In that study the hazard ratio of MC was 2.33 for ever use of estrogen, 2.12 for combined estrogen and progestin preparations, and 1.42 for progestin concerning MHT [13]. Consequently, a varying effect of different hormone preparations must be taken into consideration.

When it comes to the other factors that could influence the hormone levels no significant results were observed. Endometriosis was reported among 33 (8.6%) of the participants. This is in line with the reported prevalence in the background population, i.e., 10% [19]. As many as 135 patients (35.2%) stated that they had undergone bilateral oophorectomy. Since this percentage is higher than anticipated the patient files in Malmö, Lund and Linköping were double checked and it seems as if the number is correct. Regardless of this, in view of the fact that it is a retrospective study, we don’t know whether the patients had been operated before or after menopause and we don’t know if all operations really were bilateral. In view of these circumstances the outcome must be interpreted with caution.

One strength of the present study is that the patients exposed to certain medicines or conditions were compared with individuals from the same cohort simultaneously and with the same questions. However, the present study also has some limitations. As all study participants had MC, it was not possible to estimate the risk for disease onset. The fact that we do not have access to the timing of medication and different hormone affecting conditions make any estimation on the effect on disease onset impossible why the present study is focused on the disease course. In many cases the time span from medication and/or surgery until the questionnaire was distributed was long. Consequently, some patients could not remember how long time they had been treated with contraceptives or MHT, moreover, some did not know the exact names of their medicines hampering further analyses. Accordingly, the risk of recall bias is given in this retrospective study design. The treatments administered vary considerably, and as a result, the impact of different hormonal therapies on hormonal balance also differs accordingly. Furthermore, the number of participants is small why sampling errors could occur.

In conclusion, this study could confirm the previously known association with smoking but also with celiac disease but also revealed a negative association with oophorectomy, while the results for oral contraceptives and HRT were insignificant. The observation that oophorectomy could influence the disease course indicates that the hormonal levels could still have an effect on MC despite the lack of significant impacts of oral contraceptives and MHT in the present study. These findings highlight the need to consider the varying effects of different hormonal factors on the disease course. Further research is warranted to explore this phenomenon in greater detail.

## Electronic supplementary material

Below is the link to the electronic supplementary material.

Supplementary Material 1

## Data Availability

The data that support the findings of this study are not openly available due to reasons of sensitivity but are available from the corresponding author upon reasonable request. Data are located in controlled access data storage at Skåne university hospital, Malmö.
