# Supplementary material for: The disease course in microscopic colitis may be influenced by hormonal factors
Source: BMC Gastroenterol. 2025 Jun 19;25:438. doi: 10.1186/s12876-025-04083-8 (PMC12180190; doi:10.1186/s12876-025-04083-8)
Supplement: Supplementary file 1 — Supplementary Material 1 [file 12876_2025_4083_MOESM1_ESM.docx]

**Id no** (Fills out by the researcher) **__________ Date ____________**

**Study about the role of hormones in microscopic colitis**

You have or had an inflammation in the gut leading to diarrhoea. In a new study we wish to ask you a few questions at one occasion. If you have difficulties to remember just answer the best you can. If you don’t know go to the next question. We appreciate your efforts in answering this questionnaire.

**Many thanks for you participation that helps us to better understand how hormones may be related to your disease!**

**Basic data:**

Name: ______________________________________________________

Personal id number: ___________________________________________

Weight (kg): _____________

Length (cm): _____________

Do you smoke?

- Yes
- No

Have you been smoking?

- Yes
- No

If you smoke or had smoked – how many cigarettes do/did you smoke in average per day?

_______________

At what age did you start smoking? _______________

For how many years have you been smoking? _______________

If you have stopped – how many years ago was that? _______________

Have you been diagnosed with coeliac disease?

- Yes
- No

**Questions about the microscopic colitis:**

Do you remember how old you were when you were diagnosed with microscopic colitis?

(age) _______________

How long time did you have continuous symptoms before the diagnosis?

(Estimate in months or years) _______________

Have you been prescribed medication for the disease (Budesonide, Entocort, Budenofalk, Cortiment)?

- Yes
- No

How many short rounds have you been prescribed (approximately 6-8 w) during your disease course? _______________

If you have been prescribed the medication for longer periods to avoid flare-ups, how many months or years have you been taking it? _______________

How has the disease course been the last five years?

- No symptoms and no treatment (Budesonide, Entocort, Budenofalk, Cortiment).
- Remitting disease demanding treatment that has been efficient.
- The symptoms are under control, but it is necessary with continuous treatment.
- The symptoms are continuous despite treatment.

**Questions about hormones:**

How old were you when you got your first menstruation? (Age) _______________

Have you noticed changed toilet habits during menstruation?

- Yes
- No

If yes, what type of change? (Otherwise go to the next question)

- Looser
- Harder

If yes, when does this change occur?

- Before the menstruation
- During the menstruation
- After the menstruation

Have your menstruations been regular?

- Yes
- No

Have you been suffering from polycystic ovary syndrome (PCOS)?

- Yes
- No

Have you been suffering from endometriosis?

- Yes
- No

Do you use or have you been using oral contraceptives or any other preventive medication with hormonal effect?

- Yes
- No

If yes, mark which one(s) you have been using:

*P-pills, hormone spiral, hormone injection, hormone rod, p-rings*

(OBS Copper spiral does not contain any hormones so then you should not mark “spiral”)

For how long did you use the different methods? (Specify – if possible – type and duration)

____________________ _______________

____________________ _______________

____________________ _______________

Do you remember the names?

____________________

____________________

____________________

Have you been giving birth?

- Yes
- No

If yes, how many children have you been giving birth to? _______________

Have you been treated with ”IVF” or any other hormone stimulating measure to be pregnant?

- Yes
- No

Have you been operated and got your ovaries removed?

- Yes, one or just a a part
- Yes, both
- No

If yes, how old were you when you were operated? (Age) _______________

If you have not yet come into menopause, state it here ___ and the questionnaire is finished.

**Many thanks for your participation!**

**For you who have come into menopause:**

How old were you when you got your first symptoms indicating menopause? (Age) _______________

How old were you when the menstruations ceased? _______________

Do you medicate with any hormonal substitution for menopausal symptoms now?

- Yes
- No

If yes, indicate which one/ones: Tablets, plasters, vagitorium

Can you sate the name/names? ____________________ ____________________

For how many years have you been taking this medication? _______________

Do you remember the name/names?

____________________

____________________

____________________

Have you been taking any hormonal substitution previously?

- Yes
- No

If yes, indicate which one/ones: Tablets, plasters, vagitorium

For how many years did you use this substitution? (Years) _______________

How old were you when you stopped? (Age) _______________

Do you remember the name/names of the medicines?

__________________

____________________

____________________

***Many thanks for your participation!***

*Do you have any questions regarding this questionnaire please do not hesitate to contact your local research contact. See below for further information. If you should have any questions regarding your disease, contact your local doctor instead.*
